# The role of point-of-care ultrasound in the assessment of pelvic urine leakage and diagnosis of urinoma

**DOI:** 10.1186/s12245-023-00571-4

**Published:** 2023-12-18

**Authors:** Asra Moradkhani, Mona Zangi, Mobin Azami, Mohammad Ghasemi-Rad, Abdolghader Pakniyat

**Affiliations:** 1 Student Research Committee, Kurdistan University of Medical Sciences, Sanandaj, Iran; 2 Disaster and Emergency Management Center of Kurdistan University of Medical Sciences, Sanandaj, Iran; 3 Department of Interventional Radiology, Baylor College of Medicine, 1 Baylor Plaza, Houston, TX USA; 4 Department of Emergency Medicine, Faculty of Medicine, Kurdistan University of Medical Sciences, Sanandaj, Iran

**Keywords:** Point-of-care ultrasound, Urinoma, Renal pelvis rupture, Case report

## Abstract

**Background:**

Urinoma, a rare condition resulting from urine leakage due to trauma to the kidney, bladder, or urethra, is typically diagnosed using enhanced computed tomography urogram with delayed imaging. This report presents two cases of urinoma likely caused by overdistention of the renal pelvis following excessive fluid intake and the presence of a ureteral stone.

**Case presentation:**

We present two cases of 36-year-old and 38-year-old patients who presented with flank pain. point-of-care ultrasound (POCUS) played a pivotal role in identifying perinephric fluid in Morrison’s space and the splenorenal space, respectively. These ultrasound findings guided further investigations, leading to definitive diagnoses via abdominal pelvic CT scans. Treatment involved prophylactic antibiotics and the successful placement of a double J stent into the renal pelvis over the wire under fluoroscopic guidance, which resulted in significant clinical improvement for both patients.

**Conclusions:**

This study demonstrates the rare occurrence of urinoma from urolithiasis, the use of POCUS in expediting diagnosis and treatment, and the importance of interpreting sonographic images in the correct clinical setting.

## Introduction

Urinoma is a rare pathological condition characterized by the extravasation of urine due to a disruption in the urinary collecting system, which can occur anywhere from the calyx to the urethra [1, 2]. This pathology can manifest in various locations, including the perirenal and retroperitoneal spaces, peritoneal cavity, pleural cavity, and even the mediastinum [3]. Initially, a urinoma may be clinically asymptomatic but can present with symptoms such as pain and lead to complications like hydronephrosis, electrolyte imbalances, ileus, and abscess formation. While tumors and stones have been reported as causes of urinoma, kidney trauma remains the primary culprit [4]. Urine leakage from the urinary tract can occur due to various factors, including penetrating trauma, nephrolithiasis, pregnancy, pelvic mass, posterior urethral valves, retroperitoneal fibrosis, and bladder outlet obstruction [5]. When a urinoma occurs from an atraumatic etiology, it is classified as a spontaneous urinoma [6]. Given the potential complications, diagnosing urinoma is critical, necessitating hospitalization in specialized services and prompt treatment. Selective diagnostic imaging techniques such as computed tomography (CT) with contrast, CT cystography, and retrograde urethrography are employed to confirm the diagnosis [7, 8]. Ultrasonography is an alternative imaging method that offers the advantage of a lower cumulative radiation dose, speed, and availability compared to CT, and comparable clinical outcomes without significant side effects in the setting of suspected nephrolithiasis [9]. This study aims to illustrate the unique aspects of two cases of urinoma resulting from spontaneous rupture of the renal pelvis due to ureteral lithiasis. We underscore the pivotal role of point-of-care ultrasound (POCUS), an imaging modality that yielded critical findings, significantly influencing patient management.

We report two cases that presented with flank pain and were admitted to a tertiary referral hospital emergency department. Neither had a relevant family or medical history, nor a prior history of renal stones or trauma. Initial physical examinations revealed flank tenderness but were otherwise unremarkable. The emergency physician performed the POCUS. The use of POCUS revealed mild to moderate perinephric fluid in both patients. Subsequent abdominal pelvic CT scans confirmed pelvic urine leakage, explaining the perinephric fluid and flank pain. After diagnosis, a management plan was implemented for each patient, followed by follow-up procedures, including repeated ultrasound examinations.

## Case 1

**Fig. 1:**
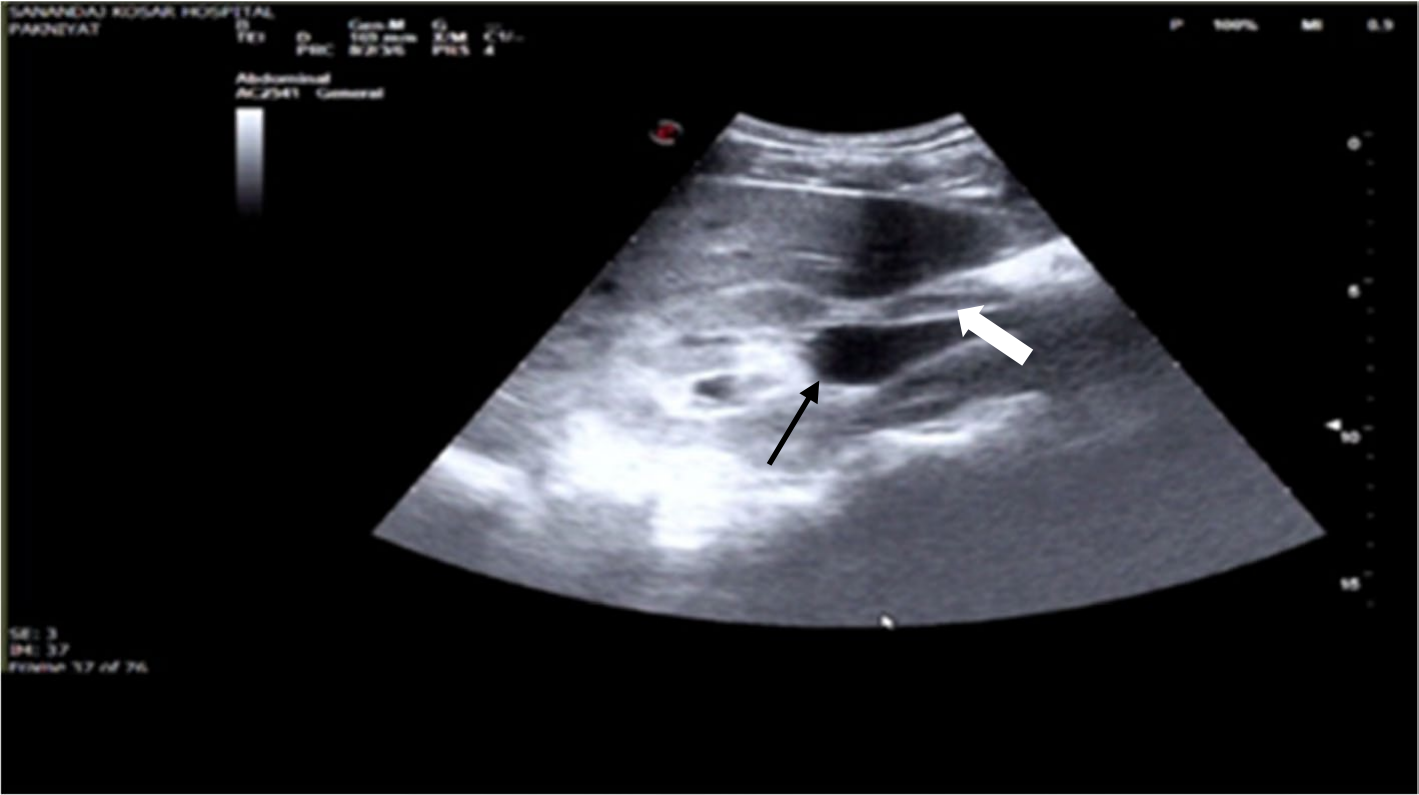
Ultrasound showing perinephric fluid and dilated ureter

**Fig. 2:**
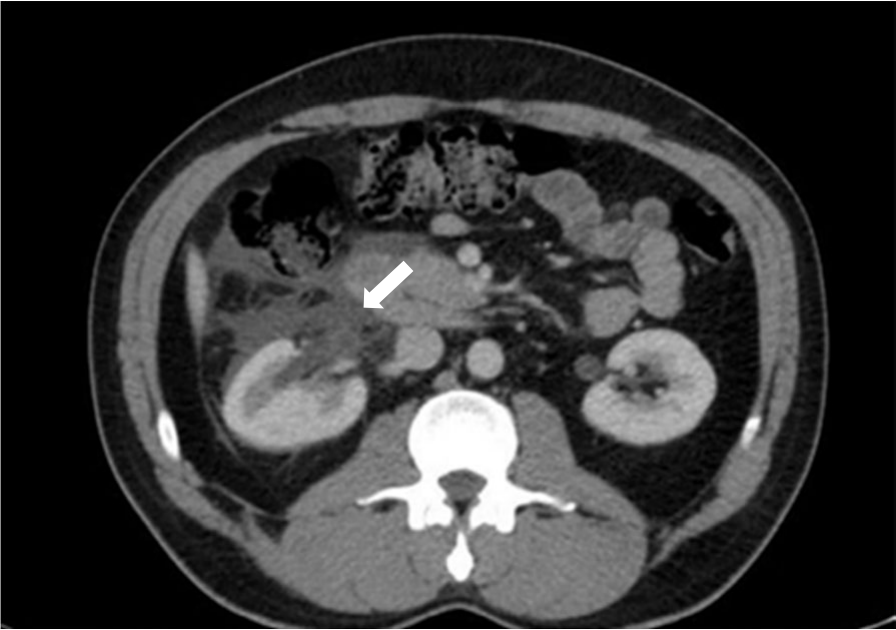
Abdominopelvic CT scan with contrast showing renal pelvis rupture

**Fig. 3:**
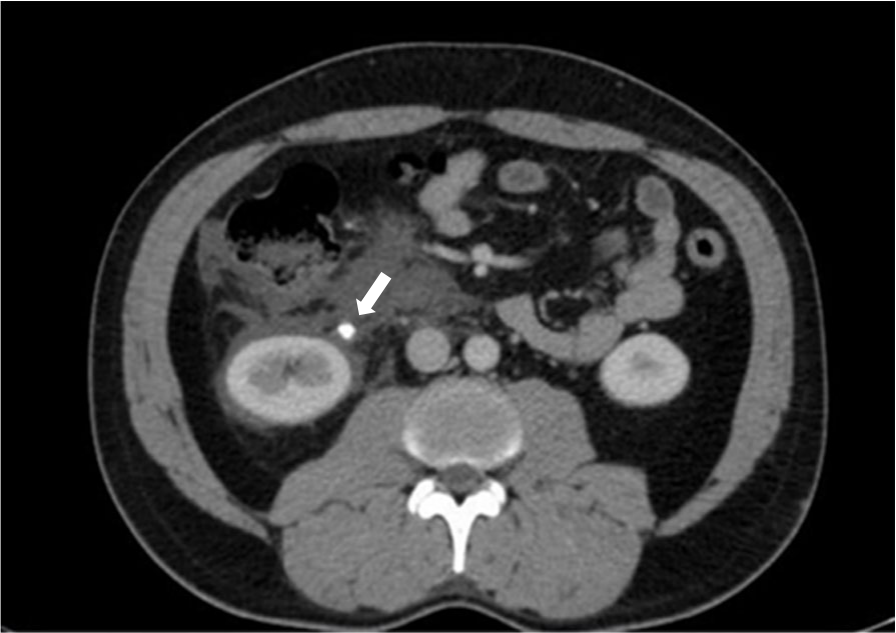
Abdominopelvic CT scan with contrast showing a small stone in the ruptured renal pelvis

A 36-year-old man presented with persistent right-side flank pain, nausea, vomiting, dysuria, and tea-colored urination. Despite consuming a traditional remedy of beer and honey and taking analgesics, his pain persisted. Physical examination revealed right abdominal tenderness and mild costovertebral angle (CVA) tenderness. Bedside ultrasound showed moderate hydronephrosis, a dilated ureter, and perinephric fluid in Morrison’s space but there was no fluid in the LUQ and suprapubic spaces (Fig. 1). A contrast-enhanced abdominopelvic CT urogram was performed, which showed renal pelvis rupture and a small stone in the ruptured renal pelvis (Figs. 2 and 3). Serological and biochemistry tests were conducted, yielding the following results:

WBC of 14,100, hemoglobin of 13.4, platelets of 207,000, creatinine of 1.5, BUN of 15, Na of 141, K of 4.2, and a blood sugar of 95.

The patient’s pain was unresponsive to narcotics, so although the urinalysis was not positive for infection, we administered 1 g ceftriaxone and 400 mg ciprofloxacin. No defect was detected in the ureter but a defect was found in the renal pelvis due to rupture. A ureteroscopy has not been done. Under guidewire guidance, a double J stent was successfully placed to bypass the defect of the renal pelvis. Treatment with ciprofloxacin 500 mg twice daily was continued. The stent was removed successfully after 1 month.

## Case 2

A 38-year-old man with severe left-side flank pain lasting for 2 days was admitted. Physical examination revealed left CVA tenderness and generalized pain on the left side of the abdomen. Laboratory results were as follows:

WBC of 15,300, hemoglobin of 15, platelets of 250,000, creatinine of 1.3, BUN of 16, Na of 138, K of 5, blood sugar of 105, Urinalysis: RBC = many, bacteria = Negative.

**Fig. 4:**
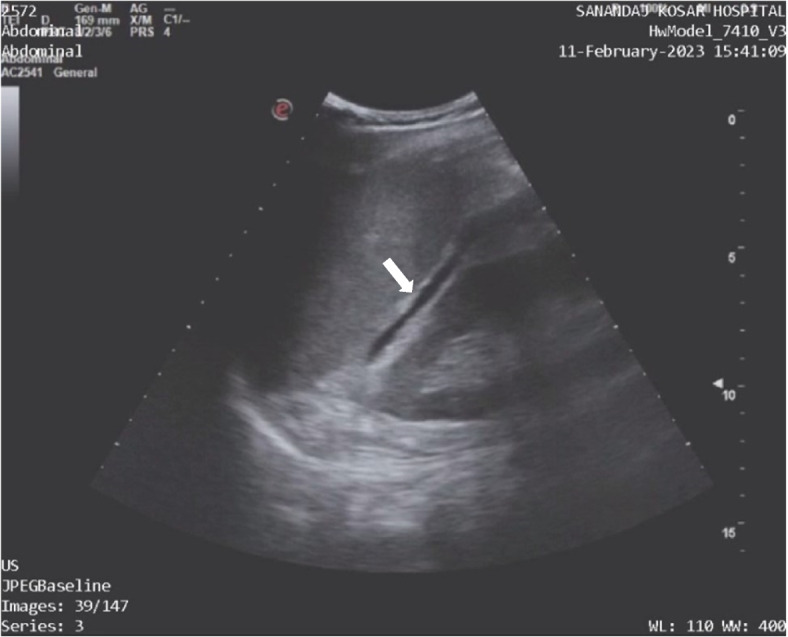
Ultrasound showing perinephric fluid

**Fig. 5:**
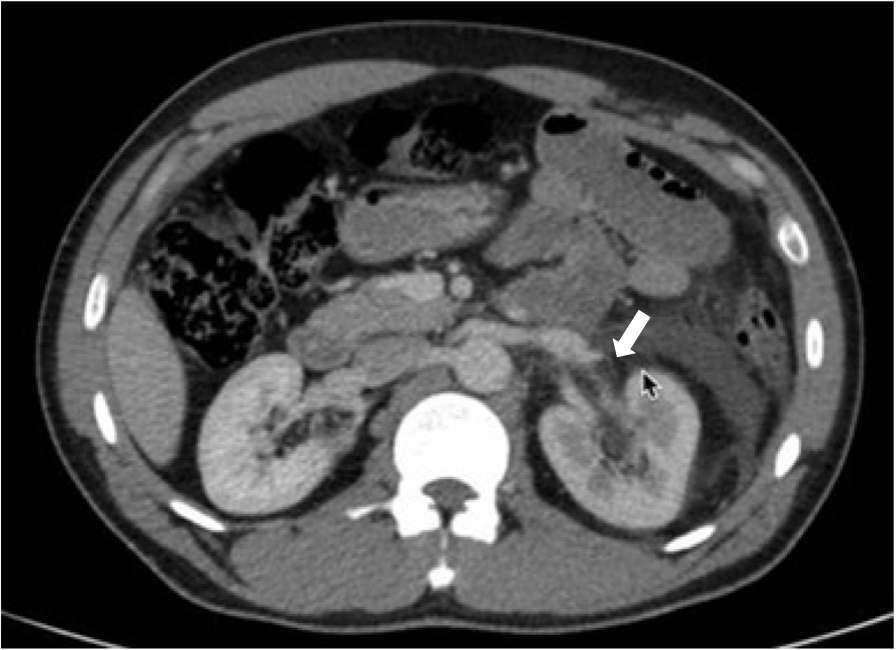
Abdominopelvic CT scan with contrast showing perinephric fluid and renal pelvis rupture

**Fig. 6:**
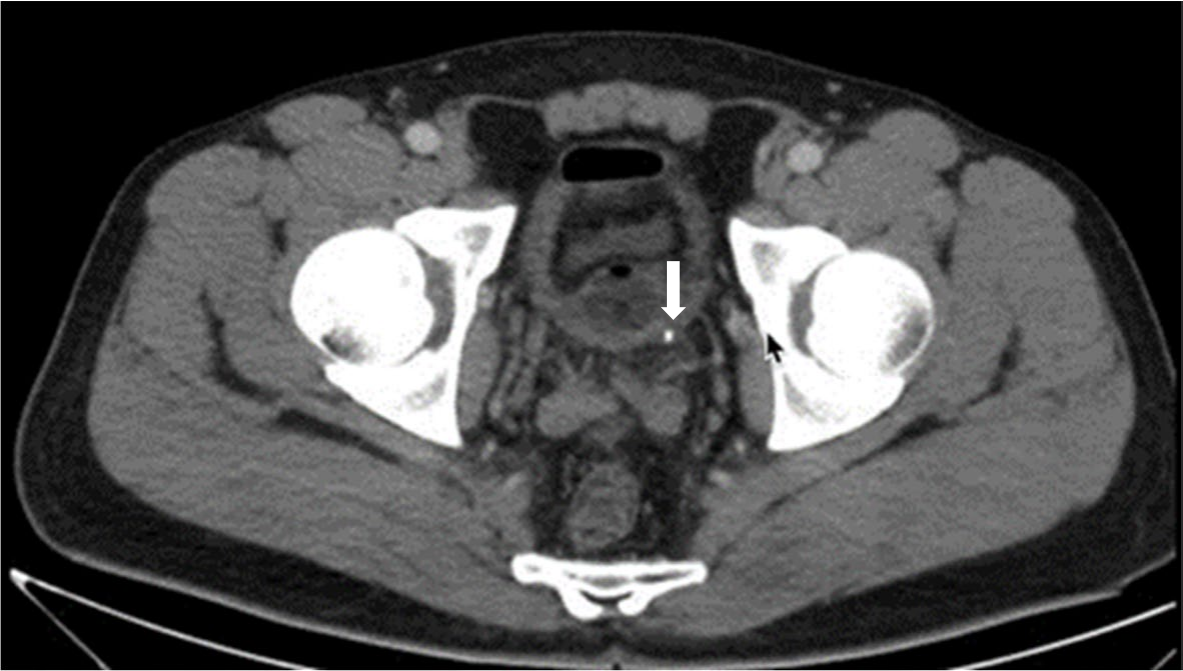
Abdominopelvic CT scan with contrast showing a small stone in the ureterovesical junction

Ultrasound revealed fluid in the splenorenal space and around the bladder and no fluid was found in other areas (Fig. 4). A subsequent abdominal pelvic CT scan showed renal pelvis rupture and perinephric fluid (Figs. 5 and 6). The patient received intravenous antibiotics. Ureteroscopy showed no abnormalities in the ureter but there was a defect in the renal pelvis. A double J stent was inserted into the renal pelvis. After 4 weeks, we removed the double J stent and the patient’s symptoms alleviated during the post-procedure monitoring phase.

## Discussion

Extravasation from any organ involved in urine formation (kidney, ureter, bladder, and urethra) can lead to a urinoma. This rare condition can be encapsulated by fibrous capsules or appear as free fluid. Kidney trauma is often implicated in urinoma formation, especially due to non-obstructive causes that lead to disruptions in the urinary collecting system. Conversely, iatrogenic injuries resulting from genitourinary, retroperitoneal, or gynecologic surgery are the main contributors to ureteral urinoma [10]. In our study, we indeed observed fluid in atypical locations for trauma cases with a positive Focused Assessment with Sonography in Trauma (FAST) exam. Specifically, in case 1, we detected fluid in Morison space accompanied by hydronephrosis, and in case 2, the fluid was found in the splenorenal space with additional left lower quadrant interloop fluid and hydronephrosis. Notably, no stones were detected in either case. Given the absence of a history of trauma; history of cirrhosis, congestive heart failure, and other causes of ascites; and the presentation of flank pain, our initial diagnoses were renal colic and urinoma, underlining the importance of utilizing POCUS in expediting diagnosis and management and interpreting sonographic evidence of free intraperitoneal fluid in the correct clinical context.

Ureteral impact stones represent a rare cause of urinoma, where the presence of a stone can lead to perforation of the ureter and subsequent urinoma formation. In our presented cases, we propose that the sudden dilation of the kidney’s pelvis due to severe diuresis following the consumption of a large amount of beer as a diuretic, combined with ureteral obstruction caused by a ureteral stone, contributed to renal pelvis rupture and urinoma formation. Studies have reported that intrapelvic pressures exceeding 25 to 75 mmHg are associated with ruptures, with the fornices being the most common site due to their thinner walls [11]. While most urinomas are small and resolve spontaneously, larger and expanding urinomas may necessitate intervention [12]. For the diagnosis of urinoma, an ultrasound examination is a valuable screening tool to assess for free intraperitoneal fluid and the cystic nature of the mass. Additionally, CT scans are alternative imaging modalities for evaluating masses within the retroperitoneum [13, 14]. Larger urinomas may demand the placement of a percutaneous drain, nephrostomy tube, or open surgery along with correction of the underlying cause. Untreated urinomas can result in serious complications, including urinary peritonitis, fibrosis, fistulae, abscess formation, and septic shock [4].

Larger urinomas are not reabsorbed and at risk of infection or sepsis. In these cases, drainage under the guidance of ultrasound or CT scan should be considered. The placement of a drainage catheter can help reduce intravesical pressure and ensure adequate drainage of the collecting system to control urine leakage. Furthermore, initiating empiric antibiotic therapy is crucial in preventing urinoma infection [7]. By facilitating early detection of abnormal fluid accumulation, POCUS plays a crucial role in identifying unusual causes of flank pain, underscoring its value in clinical practice [15, 16]. The information presented in Table 1 elucidates the occurrences of spontaneous urinoma in the renal pelvis due to ureteral lithiasis as observed in previous similar cases. These reports significantly contribute to the ongoing advancement in understanding this rare condition and emphasize the difficulties faced in managing it effectively.

**Table 1:** Details of previous similar reports of spontaneous urinoma of the renal pelvis due to ureteral lithiasis. These reports add to the growing understanding of this rare condition and the challenges in its management

Authors	Publication year	Journal	Title and abstract
S. A. Nedjim et al. [6]	Aug 2021	Radiology Case Reports	Spontaneous rupture of the fornix due to ureteral lithiasis of 3 mm caused the clinic to report the case of a 37-year-old patient who presented to the emergency department with hyperalgesia renal colic. Intense right lumbar tenderness was found on physical examination, renal function was preserved. CT scan revealed significant ureteral hydronephrosis by urinary meatus calculus and a perirenal effusion and extravasation of the PDC at a late time. The emergency therapeutic management consisted of a bypass with a double J stent
C. Thom et al. [17]	Feb 2018	The Journal of Emergency Medicine	Point-of-care ultrasound identifies Urinoma complicating simple renal colic. Three cases of unanticipated perinephric fluid collections identified initially on POC ultrasound in cases of suspected simple renal colic were presented. Concomitant hydronephrosis was also seen in each of these cases. Although the ideal management of these cases is not completely defined from the current literature, we benefit from knowing how to identify these on POC ultrasound, understanding the underlying pathophysiology, and appreciating the possibility of complications that may arise
E. Pampana et al. [11]	Dec 2013	Case Reports in Radiology	Spontaneous Ureteral Rupture Diagnosis and Treatment case of a 69-year-old woman who presented at the emergency department of our institution with severe abdominal pain. a complete laboratory evaluation was performed and abdominal contrast-enhanced CT evaluation showed contrast agent extravasation outside the excretory system without any evidence of renal calculi at basal acquisition. a double-J stent placement which was followed by complete healing of the ureter and its removal was performed 8 weeks later

## Conclusion

In conclusion, our case studies highlight the rare occurrence of urinoma due to urolithiasis and the potential role of diuresis in contributing to renal pelvis rupture. The use of POCUS was instrumental in the early detection of these cases, emphasizing its value in the diagnosis of unusual causes of flank pain. Our findings underscore the need for prompt diagnosis and treatment of urinoma to prevent serious complications. Further research is needed to better understand the pathophysiology of this condition and to refine treatment strategies.

## Patient perspective

The patients expressed relief at having a definitive diagnosis, and they appreciated the non-invasive nature of the ultrasound examination in the diagnostic process.

## Data Availability

The datasets used and/or analyzed during the current study are available from the corresponding author upon reasonable request.

## Abbreviations

FAST: Focused Assessment with Sonography in Trauma
CT: Computed tomography
CVA: Costovertebral angle
